# 

**Published:** 1892-08-27

**Authors:** 


PRICE TWOPENCE.

309, Vol. XII.—August 27th, 1892.

[Registered at the General Post Office as a Newspaper for transmission in the United Kingdom and Abroad.]

### THE HOSPITAL

WITH EXTRA NURSING SUPPLEMENT

Per Annum, 8s. 6d., post free.

Monthly Parts, 6d.; post free, 8d.

Ditto per Annum, 8s., post free.

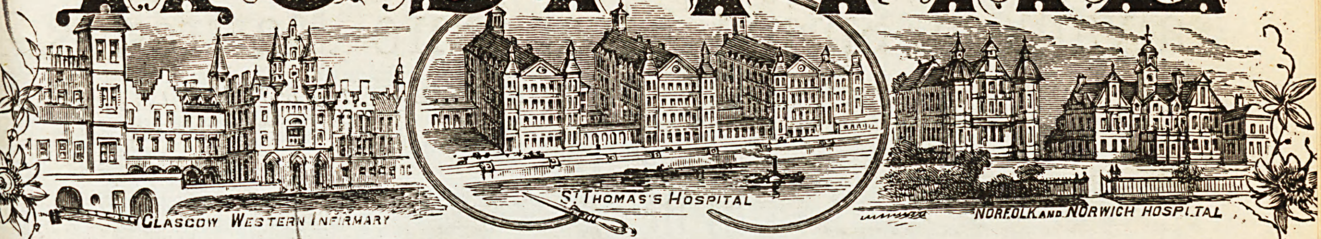

WITH EXTRA NURSING SUPPLEMENT.

No. 309. Vol. XII.] SATURDAY, AUGUST 27th, 1892 [TWOPENCE.

